# Reducing self-shading effects in *Botryococcus braunii* cultures: effect of Mg^2+^ deficiency on optical and biochemical properties, photosynthesis and lipidomic profile

**DOI:** 10.1186/s40643-021-00389-z

**Published:** 2021-04-26

**Authors:** Néstor David Giraldo, Sandra Marcela Correa, Andrés Arbeláez, Felix L. Figueroa, Rigoberto Ríos-Estepa, Lucía Atehortúa

**Affiliations:** 1 Grupo de Biotecnología, Instituto de Biología, Facultad de Ciencias Exactas y Naturales, Universidad de Antioquia UdeA, Calle 67 No. 53-108, Medellín, Colombia; 2 Grupo de Bioprocesos, Departamento de Ingeniería Química, Universidad de Antioquia UdeA, Calle 67 No. 53-108, Medellín, Colombia; 3 Max Planck Institute of Molecular Plant Physiology, Am Mühlenberg 1, Potsdam, Germany; 4 Institute of Biotechnology and Blue Development (IBYDA), University of Malaga, Campus Universitario de Teatinos s/n, 29071 Málaga, Spain

**Keywords:** Biofuels, *Botryococcus braunii*, Lipidomics, Mg^2+^ limitation, Photosynthesis, Self-shading effect

## Abstract

Microalgae biomass exploitation as a carbon–neutral energy source is currently limited by several factors, productivity being one of the most relevant. Due to the high absorption properties of light-harvesting antenna, photosynthetic cells tend to capture an excessive amount of energy that cannot be entirely channeled through the electron transfer chain that ends up dissipated as heat and fluorescence, reducing the overall light use efficiency. Aiming to minimize this hurdle, in this work we studied the effect of decreasing concentrations of Magnesium (Mg^2+^) on the chlorophyll *a* content, photosynthetic performance, biomass and lipid production of autotrophic cultures of *Botryococcus braunii* LB 572. We also performed, for the first time, a comparative lipidomic analysis to identify the influence of limited Mg^2+^ supply on the lipid profile of this algae. The results indicated that a level of 0.0037 g L^−1^ MgSO_4_ caused a significant decline on chlorophyll *a* content with a concomitant 2.3-fold reduction in the biomass absorption coefficient. In addition, the Mg^2+^ limitation caused a decrease in the total carbohydrate content and triggered lipid accumulation, achieving levels of up to 53% DCW, whereas the biomass productivity remained similar for all tested conditions. The lipidome analysis revealed that the lowest Mg^2+^ concentrations also caused a differential lipid profile distribution, with an enrichment of neutral lipids and an increase of structural lipids. In that sense, we showed that Mg^2+^ limitation represents an alternative optimization approach that not only enhances accumulation of neutral lipids in *B. braunii* cells but also may potentially lead to a better areal biomass productivity due to the reduction in the cellular light absorption properties of the cells.

**Supplementary Information:**

The online version contains supplementary material available at 10.1186/s40643-021-00389-z.

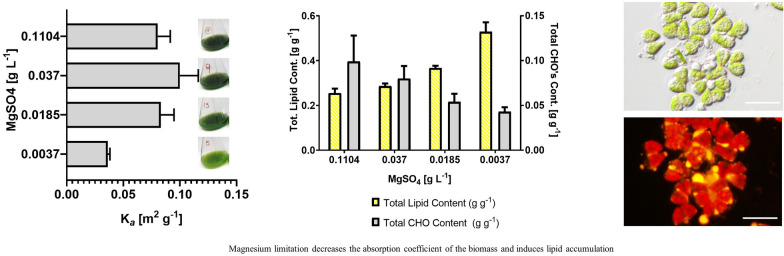

## Introduction

The global quest for developing technologies conceived to accelerate the transition from an oil-based and carbon intensive economy into renewable energy infrastructure, is passing from being a long-term scientific interest to be a top public priority due to the imminent consequences of climate change and the growing world population (IRENA 2019). Although the migration to efficient electric systems powered by renewable sources is occurring gradually during the last few years, the complete replacement of internal combustion systems is far from being fully accomplished at the scale needed to alleviate the effects of the still rising carbon concentrations in the atmosphere (IRENA 2019). In that sense, carbon dioxide (CO_2_) neutral biobased technologies represent suitable transitory alternatives for energy and raw materials production.

The use of microalgal biomass as a platform for harnessing solar energy to fix CO_2_ and generate industrially valuable compounds, has been widely studied due to a number of key advantages that had been attributed to these organisms (Choi et al. 2019). However, the scalability of microalgal bioenergy businesses is currently hindered by economic barriers related in part to the system productivity and downstream processing costs (Gifuni et al. 2019). Accordingly, many authors have proposed several strategies intending to improve the overall biomass production efficiency (Gupta et al. 2014; Vecchi et al. 2020; Takahashi 2021). One of these approaches consists in relieving the strong light attenuation of algal biomass (i.e., self-shading effects) to increase the volumetric light availability and thus improve the areal productivity (Barros et al. 2003). Some authors have evaluated artificial illumination systems based on LED technology to provide fine tunned flashing light pulses designed to maintain high photosynthetic rates without causing photoinhibition (Katsuda et al. 2006; Vejrazka et al. 2011; Fu et al. 2012). Albeit this strategy has proven to be promising, its application at large scale cultures is challenging considering the high cost of artificial illumination infrastructure and operation (Schulze et al. 2020). Alternatively, the reduction of the antenna size in the light-harvesting complexes (LHC) through the manipulation of chlorophyll synthesis or LHC assembly genes has been evaluated as a promising method (Friedland et al. 2019). Such strategy has resulted in a 40% increase in overall biomass yield for plants and algae (Perrine et al. 2012), but its implementation depends on the use of genetically modified organisms (GMO) which currently represents a significant barrier for large scale applications.

Magnesium (Mg^2+^) is the fifth most abundant element on the earth’s surface, the third most abundant ion in seawater, the second most abundant cation in cells, and is an essential element for the life on this planet. This element plays several metabolic and physiological roles, since it serves as: (i) a cofactor for more than 300 enzymes; (ii) a stabilizer of nucleic acid conformation; (iii) a ribosome structure stabilizer; and (iv) generally maintains the structural integrity of membranes in cells and organelles (Vernon and Wacker 1978; Walker et al. 1982; Hawkesford et al. 2012). In addition, Mg^2+^ is incorporated in the center of the porphyrin ring of chlorophyll molecules. Despite its importance for living organisms, the number of studies focusing on Mg^2+^ metabolism in algae and its role exerted on cellular activities is still relatively low.

There are numerous publications related to Mg^2+^ usage as a flocculating agent (Smith and Davis 2012; Schlesinger et al. 2012; Vandamme et al. 2015; Zhang et al. 2016; Ummalyma et al. 2017), but only a modest number of publications regarding the fate of chlorophyll content, photosynthetic activity or metabolite accumulation of algae cells subjected to different Mg^2+^ regimes (Finkle and Appleman 1953a; Deng et al. 2011; Gorain et al. 2013; Çakmak et al. 2014; Esakkimuthu et al. 2016; Hanifzadeh et al. 2018; Vishwakarma et al. 2019; Polat et al. 2020). Furthermore, to our knowledge there is only one previous study, where the authors measured the Mg^2+^ consumption in batch cultures of *B. braunii* SAG-30.81 cells (Sydney et al. 2010). Consequently, in this study, we evaluated a straightforward approach to reduce the level of self-shading effect of the hydrocarbon producing green alga *B. braunii* by restricting the availability of this key element in the medium. We, therefore, compared the chlorophyll content and the concomitant biomass absorption capacity of cells cultivated upon different concentrations of MgSO_4_. We additionally followed the biomass production and composition, photosynthetic performance, and conducted for the first time a comparative lipidomic analysis under these specific conditions. Our results revealed that a limited magnesium supply not only affects the biomass optical properties but also triggers lipid accumulation, provoking a significant modification of the lipid profile in *B. braunii* cells, and this approach may be used as a strategy to improve the areal productivity of the system.

## Methods/experimental

### Strain and culture conditions

*B. braunii* LB 572 was obtained from the UTEX algae culture collection. For inoculum production and maintenance, the cells were cultivated in 500 mL conical flasks at 110 rpm and continuously illuminated with cool white LED lamps (50 µmol photons m^−2^ s^−1^). Modified BG_11_ medium was used according to the following composition: NaNO_3_ 1 g L^−1^; MgSO_4_.7H_2_O 0.037 g L^−1^; CaCl_2_.2H_2_O 0.036 g L^−1^; K_2_HPO_4_ 0.093 g L^−1^; FeSO_4_ 0.0034 g L^−1^; EDTA 0.001 g L^−1^; H_3_BO_3_ 0.0028 g L^−1^; MnSO_4_.H_2_O 0.0018 g L^−1^; ZnSO_4_.7H_2_O 0.22 mg L^−1^; Na_2_MoO_4_.2H_2_O 0.39 mg L^−1^; CuSO_4_.7H_2_O 0.08 mg L^−1^; CoCl_2_.6H_2_O 0.04 mg L^−1^. The carbon source was supplied in the form of CO_2_ (30 s pulses) on a daily basis, and the temperature was not controlled but remained constant between 24 and 26 °C. To evaluate the effect of the MgSO_4_ availability on the optical properties, photosynthetic performance and productivity of *B. braunii*, the cells were cultivated over 23 days in four different media formulations. Initially, *B. braunii* was grown under the standard conditions mentioned above. When the cell concentration was approximately 1.5 g L^−1^ the culture was harvested, and the cell pellet was washed three times with BG_11_ medium without MgSO_4_ by centrifugation (12,000 rpm for 5 min) and resuspension. Finally, new cultures were started with an approximate initial cell concentration of 0.4 g L^−1^, in BG_11_ medium with four different levels of MgSO_4_.7H_2_O as follows: 0.1104 g L^−1^; 0.037 g L^−1^ (Control group); 0.0187 g L^−1^ and 0.0037 g L^−1^. All treatments were evaluated by quadruplicate and samples were taken periodically throughout the incubation time to measure culture parameters. The culture conditions were the same as previously described for maintenance cultures.

### Nutrient consumption

For nutrient consumption estimation, each culture was sampled and centrifuged (30,000*g*, 5 min, Hitachi Himac CR22N. Rotor R22A). Subsequently, the cell-free supernatant was chemically analyzed. The nitrate concentration was measured through the spectrophotometric salicylic acid method according to Cataldo et al. (1975). The total phosphate concentration was estimated spectrophotometrically using the ascorbic acid method according to Butler (1984).

### In vivo chlorophyll fluorescence measurements

The photosynthetic performance of *B. braunii* cells was assessed by measuring the in vivo chlorophyll *a* fluorescence using a Pulse Amplitude Modulation fluorometer (Junior PAM. Walz GmbH-Germany). All measurements were performed after 20 min of dark adaptation of microalgal samples withdrawn directly from the culture. The maximal quantum yield of photochemical energy conversion in the Photosystem II (PSII) was determined with dark adapted cells as ${F}_{v}/{F}_{m}=\left({F}_{m}-{F}_{0}\right)/{F}_{m}$, where *F*_0_ is the minimum level of fluorescence emitted due to the exposure to measuring light and *F*_*m*_ is the maximum fluorescence obtained with a short pulse of high irradiance actinic light (10,000 µmol photons m^−2^ s^−1^) (Cosgrove and Borowitzka 2010). To assess the cell response and adaptation to different levels of irradiance under different nutrient configurations, rapid light curves (RLC) were constructed according to (Malapascua et al. 2014). For each level of irradiance, photosynthetic parameters such as the effective quantum yield (*Y*_II_ or Δ*F*/Fm’) and quantum yield of regulated or non-regulated non-photochemical dissipation of energy (*Y*_(NPQ)_ and *Y*_(NO),_ respectively), were estimated according to Malapascua et al. (2014).

### Pigment extraction and quantification

For chlorophyll *a* estimation, the fresh biomass sample was centrifuged (30,000*g*, 5 min) and the supernatant was discarded. The cells were then resuspended in 2 mL DMSO at 60 °C, vortexed and incubated for 10 min at 60 °C. Samples were centrifuged and the supernatant was recovered and diluted to obtain an OD (optical density) below one. The absorbance at 649 nm and 665 nm were measured in a spectrophotometer (Biotek Powerwave *X*_2_*S*). The chlorophyll *a* concentration (mg L^−1^) was estimated as ${\text{ChlA}}=\left(12.47\times {\mathrm{OD}}_{665\mathrm{nm}}\right)-\left(3.62\times {\mathrm{OD}}_{649\mathrm{nm}}\right)$, which was then computed along with the amount of biomass used for the extraction to obtain the ChlA content per unit of cell mass (Griffiths et al. 2011).

### Estimation of absorption coefficients

After 23 days of cultivation, the cell suspensions were harvested and used for estimating the absorption coefficient of biomass (*K*_*a*_) using the method proposed by Grima et al. (1994) using a polychromatic light source as a reference (RGB cool white LED–6500 K). Briefly, the absorbances of different dilutions of known concentration for each sample were measured according to Lambert–Beer´s law. The *K*_*a*_ values were estimated for the aforementioned light source by dividing the slopes obtained from plotting absorbance over biomass concentration by the optical path length.

### Bright field and fluorescence microscopy

Fresh culture samples of *B. braunii* were observed with a microscope Nikon Eclipse 80i using a Nikon DS-Fi1 camera and Differential Interference Contrast (DIC). The IntensiLight C-HGFI Fiber Illuminator system was used for fluorescence observations of chlorophyll *a* (autofluorescence), and the neutral lipids were visualized after staining the cells with Nile Red as indicated by Alemán-Nava et al. (2016).

### Lipid extraction and quantification

Total lipid content in the biomass was estimated gravimetrically. Hence, 50 mL of each cell suspension was concentrated by centrifugation (20,000*g*, 5 min, 4 °C—Hitachi Himac CR22N. Rotor R15A) and rinsed with deionized water. The washing step was repeated twice. The concentrated cell pellet was frozen with liquid nitrogen and finely macerated with mortar and pestle. The treated biomass was then mixed with 10 mL of hexane:isopropanol (3:1), homogenized by vortexing and centrifuged (10,000 rpm, 5 min, 4 °C). The supernatant containing the lipophilic extract was transferred to a glass vial and the extraction procedure was repeated twice using the residual biomass. The lipophilic extracts were recovered and dried in a rotatory evaporator (Buchi R215). The total lipid content was calculated as the ratio between the weight of the dry lipophilic extract and the total biomass used for the extraction.

### Thin-layer chromatography

Total lipid extracts were analyzed by thin-layer chromatography (TLC) to qualitatively compare the distribution of lipid pools among different treatments. The dried lipid extracts were diluted in hexane (1–5 mL according to the extract weight to achieve an approximately similar lipid concentration in all samples) and 10µL of the dilution was loaded onto activated 10 × 12 cm F_254_ silica plates (160 °C, 30 min). The mobile phase was Hexane/diethyl-ether/acetic acid (45:5:0.5 by volume). The plates were revealed under UV light.

### Lipidome analysis

Lipidome analysis was performed by UPLC–MS (Acquity UPLC System) for samples taken at 23 days after the start of the incubation, according to the procedure published by Bromke et al. (2015). The mass spectra were acquired using an Orbitrap mass spectrometer and were processed with the Xcalibur™ and Refiner MS 7.5 (Genedata Expressionist^®^) software. The validation of the lipids identified was made by comparison with an in-house library. The output contained a list of features associated with the intensities of peaks. Features naturally containing the heavy carbon isotope (^13^C) were removed from the data set. The output data was normalized to the dry amount of sample used for the analysis. In the LC–MS approach used here we provide, for each feature, the combined mass of the acyl chains attached to a particular head group; thus, each lipid species is identified by the abbreviation of the lipid class, followed by the sum of the carbon atoms of the acyl chains and the unsaturation degree. As it is possible for lipids with multiple acyl chains to have different combinations of fatty acids (FA) sharing the same overall mass, the individual acyl content was not determined directly. For the statistical analysis the webserver MetaboAnalyst (Chong et al. 2019) was used. The data were auto-scaled and normalized. Significant differences were determined by performing a Kruskal Wallis test (Chong et al. 2019). To visually explore differences in the distribution of lipid profiles among nutritional schemes a principal component analysis (PCA) was carried out (Chong et al. 2019). To further investigate the patterns of the lipid species that changed across the groups of samples, heatmaps were built based on the calculated lipid ratios among the cells subjected to the different media formulations and the control samples.

### Carbohydrate extraction and quantification.

For total intracellular carbohydrates (in-CHOs) quantification, the defatted biomass residue was weighted and boiled with deionized water for 10 min. The dissolved in-CHOs were then separated from the biomass pellet by centrifugation (30,000*g*, 10 min). The total in-CHOs concentration was determined following the phenol/sulfuric acid method proposed by Masuko et al. (2005).

### Statistical analysis

All data were expressed as mean ± SD coming from four independent replicates. The one-way or two-way analysis of variance (ANOVA) was performed to analyze the significance at the level of *p* < 0.05, and the Tukey’s test was used for multiple comparison analyses.

## Results and discussion

### Growth and biomass composition

In the present study, we evaluated the response of *B. braunii* cells cultivated in medium with different Mg^2+^ levels. We followed the evolution of growth, biochemical and photosynthetic parameters along a 23-day period and performed a comparative lipidomic analysis with the biomass of each tested condition.

**Fig. 1:**
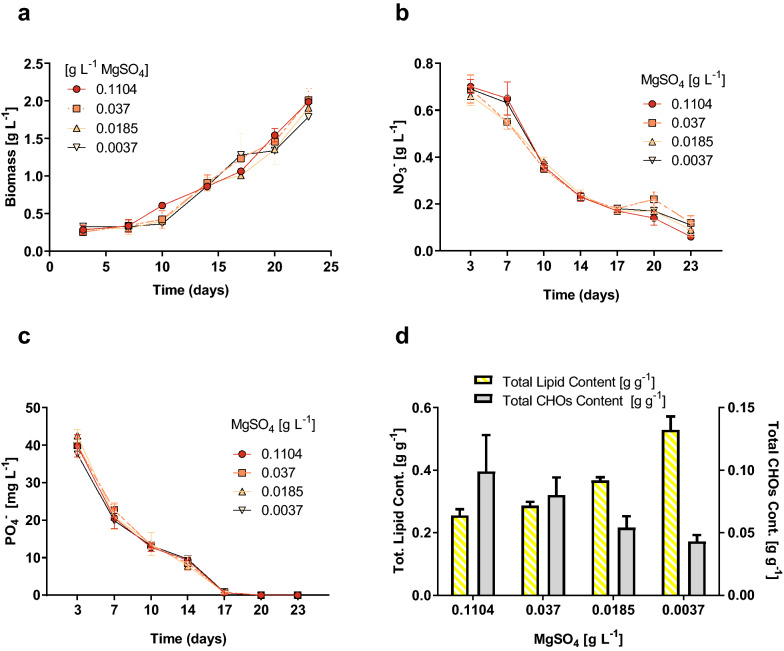
Culture parameters evaluated in *B. braunii* cultures incubated with different MgSO_4_ concentrations. **a** Growth kinetics expressed as dry cell weight; **b** dissolved nitrate concentration; **c** dissolved phosphate concentration; **d** total lipid and intracellular carbohydrates (CHOs) fraction of biomass. Data shown correspond to the means ± SD of 4 biological replicates

The results suggested that under the tested concentrations the availability of Mg^2+^ did not cause significant changes in biomass production nor macronutrient consumption during the course of the cultivation period (Fig. 1a–c) similar to the observations previously reported for *Chlorella vulgaris* (Ben Amor-Ben Ayed et al. 2015) and *Scenedesmus* sp. (Ren et al. 2014). However, it is important to note that an opposite trend has been reported for other microalgae strains (Finkle and Appleman 1953a; Gorain et al. 2013). This might indicate that *B. braunii* cells are efficient in recycling and absorbing this microelement under limiting conditions. Conversely to biomass production, the remaining cellular parameters were affected by the availability of this cation as discussed next.

The level of Mg^2+^ showed to have a clear impact on carbon biomass distribution, since the lipid:CHOs ratio varied significantly across the groups (Fig. 1d). Whereas the CHOs content of the biomass decreased with lower magnesium concentrations, the total lipids showed an opposite tendency. The lowest Mg^2+^ dose led to a total CHOs content of 4.3 ± 0.5%, which was significantly lower (*p* < 0.05) compared to the control samples (8.0 ± 1.4%) and the cells grown under the highest Mg^2+^ level (9.9 ± 2.9%). Conversely, the lowest Mg^2+^ concentration triggered the accumulation of nearly 53% of total lipids, which was almost twice the lipid content measured in the control treatment that accounted for 28.6% lipids (*p* < 0.001). The results of previous studies on lipid production in Mg^2+^ limited algal cells do not follow a consistent tendency. Polat et al. showed that Mg^2+^ deprivation induced a total lipid accumulation up to 45% DCW in *Auxenochlorella. protothecoides* (Polat et al. 2020), whereas Gorain et al. (2013) found that *C. vulgaris* and *Scenedesmus obliquus* reached similar lipid contents than the control cultures subjected to similar conditions.

Although there is a substantial variation in the total lipid contents reported in the literature for *B. braunii*, the typical interval for standard conditions ranges from 25 to 40% (Yoshimura et al. 2013; Ferreira et al. 2019). Yet, increased lipid accumulation is commonly associated with specific stress conditions. Some authors have found that nitrogen limitation up-regulates the lipid accumulation in *B. braunii* cells (Zhila et al. 2005a, b; Choi et al. 2011; Cheng et al. 2014). Nevertheless, this higher accumulation is usually achieved at expense of biomass production, which might represent an overall decline of the lipid productivity. Moreover, the lipid accumulation induction is normally implemented as a two-step process consisting of a biomass production period, followed by a stress-related accumulation stage (Rodolfi et al. 2009; Xia et al. 2013). In contrast, the results of this study revealed that even under severe magnesium limitation, the biomass production is not impaired despite the higher lipid accumulation. This fact represents an important operational advantage in the sense of facilitating the implementation of continuous modes of operation schemes (Zhou et al. 2014). Besides the changes in the lipid accumulation capacity, a qualitatively TLC analysis showed that the availability of magnesium also has a clear influence on the lipid profile of *B. braunii* cells (Additional file 1: Fig. S2) as the neutral lipid fraction showed an augmented abundance for the lowest Mg^2+^ cultures over the other Mg^2+^ regimes. To further investigate with more detail the changes on the lipid profiles, we conducted a lipidomic analysis, the results of which are discussed below.

### Comparative lipidomic analysis

**Fig. 2:**
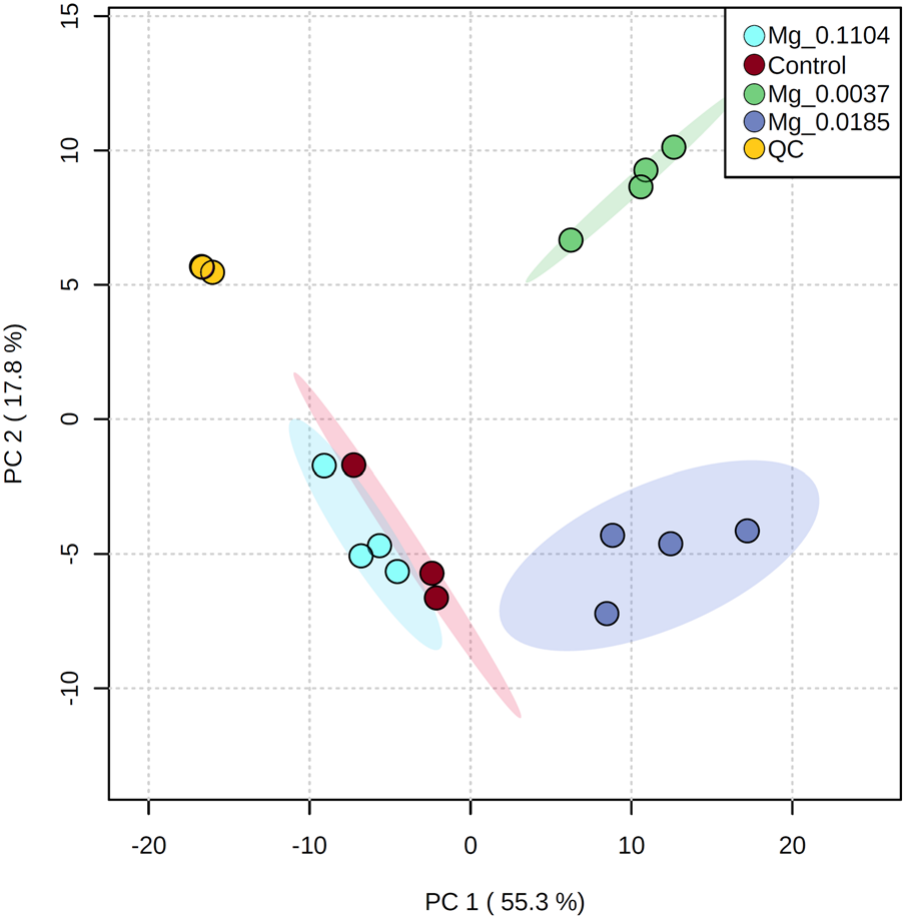
Scores plot for a principal component analysis (PCA) of *B. braunii* cells cultivated under different Mg^+2^ regimes. PC 1 and PC 2 explain the 55.3% and 17.8% of variation, respectively (cumulative 73.1% of variation explained by the model)

**Fig. 3:**
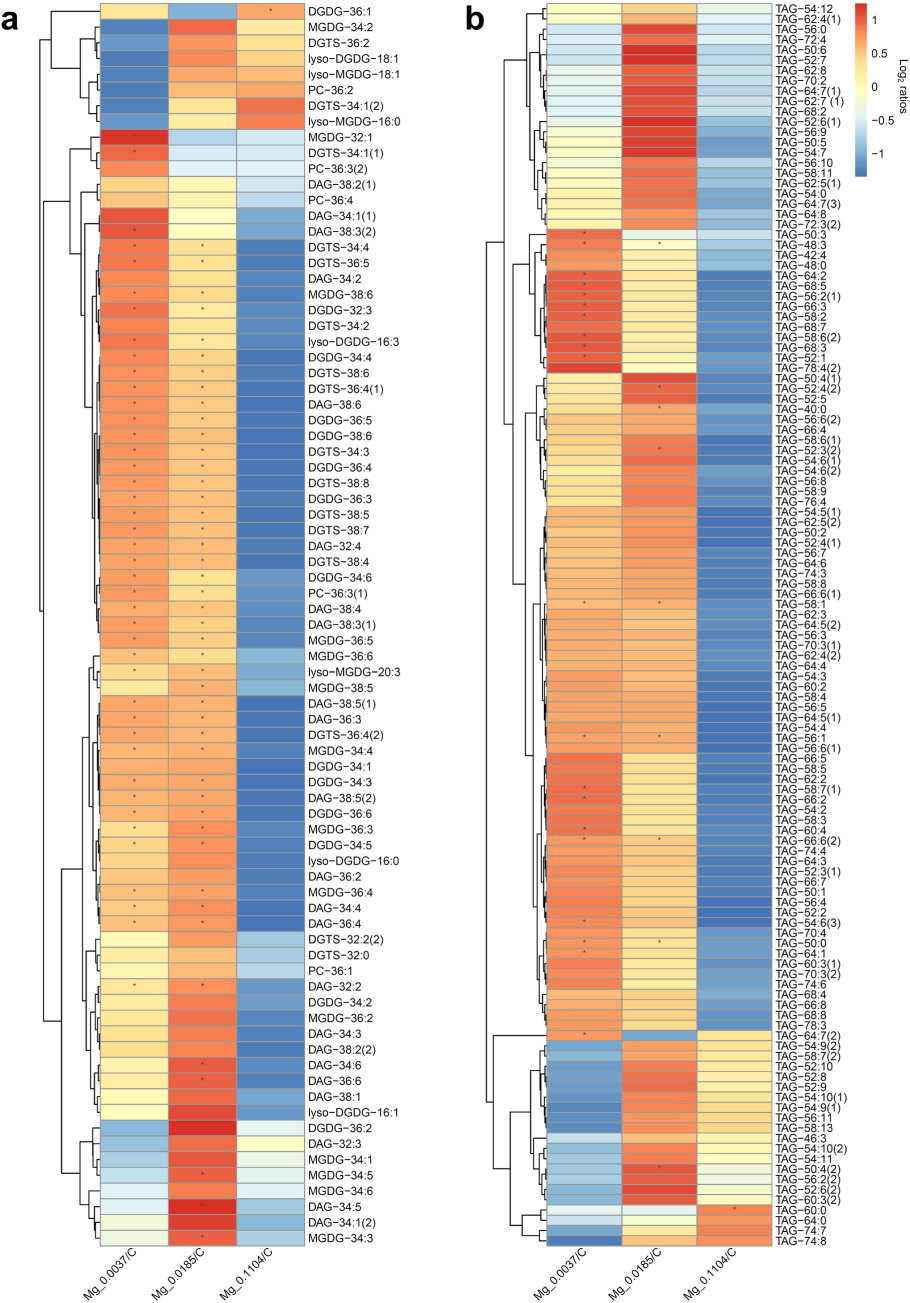
Heatmaps of the log_2_ transformed lipid abundance ratios of *B. braunii* cells under different Mg^+2^ regimes, showing. **a** DAGs, and polar lipids and **b** TAGs, distributions. The lipid species are identified by the abbreviation of the lipid class, followed by C:N, where C is the total number of carbons in acyl chains, and N is the total number of double bonds, which in some cases also includes a number in round brackets that indicate the isomers. The lipids with a higher-than two-fold change are marked with an asterisk. DAG, diacylglycerol; MGDG, monogalactosyldiacylglycerol; DGDG, digalactosyldiacylglycerol; DGTS, diacylglyceryl-N,N,N-trimethylhomoserine; PC, phosphatidylcholine; TAG, triacylglycerol

We performed LC–MS-based lipidomics profiling to characterize the differential impact of various Mg^2+^ regimes on *B. braunii* neutral and structural lipids biosynthesis. The lipidome analysis led us to identify a total of 223 lipid species, classified into several lipid classes, namely, diacylglycerol (DAG), digalactosyldiacylglycerol (DGDG), diacylglyceryl trimethyl homoserine (DGTS), monogalactosyldiacylglycerol (MGDG), phosphatidylcholine (PC) and triacylglycerol (TAG). A principal component analysis (PCA), performed on all these lipid groups, demonstrated that a high level of variation in the lipidome (55.3%) among treatments can be explained by the first component (PC1; Fig. 2). The PCA showed that Mg^2+^ levels can influence *B. braunii* lipid composition. Therefore, the lower the Mg^2+^ concentration, the lipid composition becomes significantly different from the control (Fig. 3). These results are also in accordance with the TLC analysis (Additional file 1: Fig. S2).

The differential lipid:CHOs ratio described in the previous section was also reflected in the lipidome results. That is, in *B. braunii* cells with the highest Mg^2+^ level (0.1104 g L^−1^), DAG underwent a reduction as for the control, while the opposite occurred in cells grown with the lowest Mg^2+^ levels (0.0185 and 0.0037 g L^−1^) (Fig. 3a). For the structural lipids (i.e., the galactolipids DGDG and MGDG, and the betaine lipid, DGTS), we observed that at the highest Mg^2+^ level these lipid species exhibited a reduced ratio abundance or just marginal increases of a few species. Conversely, under the lowest Mg^2+^ levels an increased abundance of the aforementioned lipids was observed, with some exceptions (Fig. 3a). In all samples, only a few phospholipids (i.e., PC) showed elevated abundance in most cases. However, given their low abundance compared to DGTS, it is probable that they were not fulfilling a structural role in *B. braunii* cells; thus, their exact function remains unclear. Regarding the TAGs, we observed that the cells under the highest Mg^2+^ level, showed a trend of TAG reduction (Fig. 3b). In contrast, at the lowest Mg^2+^ levels, the abundance of most TAGs increased, with the exception of the Mg^2+^− 0.0037 treatment, where around 13% of the identified TAGs may have suffered degradation as their ratio abundance was decreased in comparison to the control, corresponding mostly to polyunsaturated TAGs. Despite this apparent TAG degradation, we also noticed that a greater number of TAGs exhibited a twofold increase or even larger compared to the control samples (Fig. 3b). From these results, we can underline that *B. braunii* cells grown with the highest Mg^2+^ level underwent an overall reduction in all lipid classes (Fig. 3a, b). However, the question remains whether the reduced lipid content may be attributed to the down regulation of the lipid biosynthetic pathways and the reapportionment of metabolic resources towards CHOs accumulation or alternatively could be the result of a diminished availability of energy carrier (i.e., ATP, NADH and NADPH), which are essential resources for lipid biosynthesis.

Excessive amounts of Mg^2+^ in the medium causes increases in the chlorophyll content of cells (Finkle and Appleman 1953b; Hanifzadeh et al. 2018) which can then decrease light diffusion into the culture and thereby diminish culture productivity (Mitra and Melis 2008; Hanifzadeh et al. 2018). This may lead to the preferential accumulation of metabolic products with lower energetic and reductant demands compared to lipids (i.e., CHOs). Alternatively, conditions that result in a surplus of reductant, such as when microalgae cultures are exposed to suitable irradiances during nutrient deprivation, can result in diminished photosynthetic activity, with the flow of fixed carbon re-routed to the accumulation of more energy-dense metabolic products, e.g., lipids (Rodolfi et al. 2009).

We also observed that the treatments, where cells were grown under the mid-low Mg^2+^ levels did not trigger lipid remodeling responses, as there was not apparent reduction on structural lipids. One exception might be the treatment with the lowest Mg^2+^ level (0.0037 g L^−1^) as several MGDGs were reduced, which may suggest an incipient lipid remodeling response. However, further evidence is necessary to determine to what extent lipid remodeling may contribute to neutral lipid accumulation in this treatment. Therefore, we could argue that de novo synthesis was likely the prevailing mechanism for structural and neutral lipid accumulation. The capacity for de novo lipid synthesis seems to be a characteristic of some oleaginous microalgae, which when subjected to growth-limiting stress conditions such as nutrient deficiency channel excess carbon and energy towards storage lipids (i.e., TAGs) (Shifrin and Chisholm 1981; Rodolfi et al. 2009; Guarnieri et al. 2011; Klok et al. 2013; Simionato et al. 2013).

Micro- and macro-nutrient composition play a decisive role in microalgae growth. Mg^2+^ is not an exception, since microalgae cells show varied responses to Mg^2+^ status concerning the accumulation and distribution of biomass components. Studies, where Mg^2+^ surplus is provided, have shown an enhanced lipid (Gorain et al. 2013; Esakkimuthu et al. 2016) and/or biomass accumulation (Finkle and Appleman 1953a; Gorain et al. 2013). Conversely, there are reports, where conditions of Mg^2+^-limitation improved lipids accumulation (Deng et al. 2011; Çakmak et al. 2014; Hanifzadeh et al. 2018; Vishwakarma et al. 2019; Polat et al. 2020). Therefore, these results must be viewed with caution, as the relation among biomass and lipids content is not necessarily linear to Mg^2+^ levels (Gorain et al. 2013; Esakkimuthu et al. 2016; Polat et al. 2020). The later implies that there is a species-specific Mg^2+^ optimum level to achieve the maximum growth and/or lipid accumulation (Gorain et al. 2013; Hanifzadeh et al. 2018; Vishwakarma et al. 2019; Polat et al. 2020), which may also depend on a complex relationship between Mg^2+^ and other nutrients present in the growth medium (Walker 1994; Vishwakarma et al. 2019). This is also supported by our results, since we observed that *B. braunii* lipid accumulation varied according to Mg^2+^ concentration, with maximum accumulation at the lowest Mg^2+^ level tested.

In our study we did not evaluate the effect of complete Mg^2+^ starvation in *B. braunii* cells due to its limited feasibility in continuous cultures; hence, we cannot determine if Mg^2+^ deprivation may further enhance lipid accumulation. However, there is evidence that for some microalgae, a complete elimination of Mg^2+^ from the medium stimulates lipid accumulation at the expense of a drastic reduction of cellular growth (Gorain et al. 2013; Esakkimuthu et al. 2016; Polat et al. 2020), which negatively impacts lipid productivity. Moreover, Mg^2+^ excess can negatively impact growth and lipid production (Gorain et al. 2013; Esakkimuthu et al. 2016; Vishwakarma et al. 2019), since it becomes a stressor agent, apart from unnecessarily increasing production costs when it comes to commercial-scale cultures. (Gorain et al. 2013; Çakmak et al. 2014; Esakkimuthu et al. 2016; Hanifzadeh et al. 2018).

### Effect of magnesium availability on the optical properties of B. braunii:

**Fig. 4:**
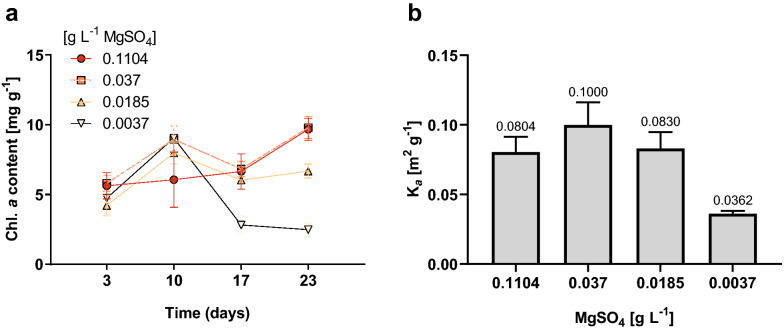
Pigment content and optical properties of *B. braunii* grown with different MgSO_4_ concentrations. **a** Chlorophyll *a* content of biomass at different time points; **b** absorption coefficient of the biomass (*K*_*a*_) after 23 days of incubation estimated for a polychromatic RGB white LED light source. Data shown correspond to the means ± SD of 4 biological replicates

Nearly 75% of the solar energy absorbed by photosynthetic organisms can be dissipated by non-photochemical routes, since photons are captured by the LHC one order of magnitude faster than the slower steps of the electron transport chain (Friedland et al. 2019). Consequently, one of the strategies proposed by some authors to improve the biomass productivity of microalgae relies on the alteration of the cellular light absorption capacity to reduce the self-attenuation effect and increase the light availability in the cell suspensions (Barros et al. 2003). An approach in this regard encompasses the reduction of the antenna size of the LHC’s by a down-regulation of proteins associated with its assembly or by inducing a decline in the activity of the chlorophyll-synthesizing enzymes (Friedland et al. 2019). However, this option entails the use of genetically modified organisms, which hampers its application and scalability, especially in open production systems. Alternatively, in this work we proposed the alteration of cellular light absorption by constraining the availability of magnesium in the culture medium aiming to diminish the levels of chlorophyll synthesis and, therefore, attenuate the absorption capacity of photosynthetically active light per cell. The results indicated that *B. braunii* cells underwent an important change on its light absorption features when they were cultivated with the lowest Mg^2+^ concentration. The chlorophyll content did not show drastic differences across all treatments during the first 10 days of culture when the cell densities were low (Fig. 4a); from that point, the chlorophyll *a* content dropped to 2.5 ± 0.5 mg g^−1^ by day 23, in cultures with 0.0037 g L^−1^ MgSO_4_, which was significantly lower compared to all other treatments (*p* < 0.05). At the end of the incubation time, the cell suspensions corresponding to this treatment showed a lighter green color compared to the other groups, despite of having a similar cell concentration (Additional file 1: Fig. S2). Similarly, the cells with the lowest Mg^2+^ levels displayed a lighter green color than the other treatments when observed under the microscope (Additional file 1: Fig. S3). The reduction in the chlorophyll content has been consistently documented in photosynthetic organisms upon Mg^2+^ shortage conditions (Finkle and Appleman 1953a; Volgusheva et al. 2015; Ben Amor-Ben Ayed et al. 2015) which is consequent with the key role of this divalent cation in the biosynthesis of this pigment, since it binds to the chlorin cycle in active chlorophyll molecules (Finkle and Appleman 1953b).

**Fig. 5:**
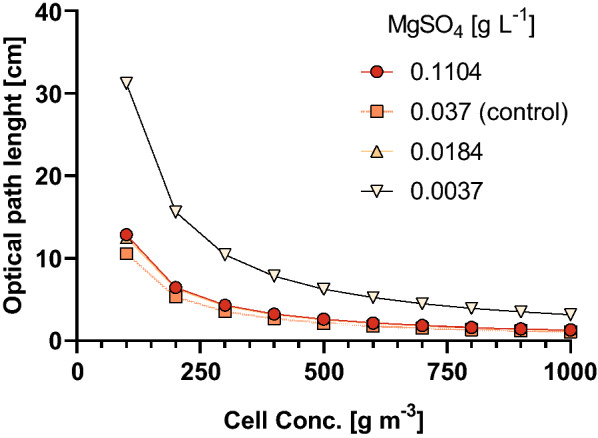
Estimation of the optical path length as a function of the cell concentration required to develop a light attenuation profile consisting of an incident irradiance of 1050 µmol m^−2^ s^−1^ at the surface of the culture and an irradiance value in the bottom (darkest zone) equivalent to the compensation irradiance (*E*_*k*_) for the cells cultivated with different MgSO_4_ concentrations. *E*_*k*_ values for each group were calculated using the model of Eilers and Peeters, 1988 employing the ChlA Rapid Light Curves data. A horizontal planar cultivation system irradiated with perpendicular direct polychromatic cool white LED light was assumed

As expected, the difference in pigment content also had a direct effect on the biomass absorption features, demonstrated as a 2.3-fold reduction in K_a_ in cultures amended with the lowest Mg^2+^ concentration compared to the control (Fig. 4b). As a consequence, the decline in light absorption capacity reduces energy supply into the electron transfer chain, and also increases the risk of photoinhibition, but the overall system productivity could potentially be improved by higher light availability resulting from the reduction in the self-shading effects, upgrading of the average irradiance in the liquid (Grima et al. 1994). In this sense, we can hypothesize that the areal biomass productivity may be improved in cultures with lower Mg^2+^ levels, opening the possibility to have greater optical path lengths for certain incident irradiance values, as depicted in the simulation presented in Fig. 5. This hypothesis, therefore, remains open to be tested in actual production conditions.

**Fig. 6:**
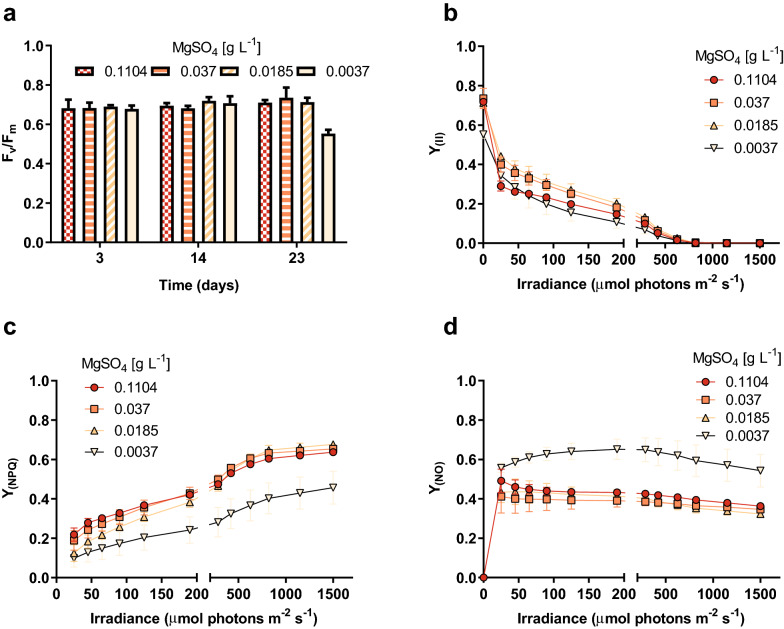
Photosynthetic parameters measured with dark adapted *B. braunii* cells. **a** Maximum quantum yield of PSII photochemistry (*F*_*v*_/*F*_*m*_); **b–d** are the rapid light curves for *Y*_II_, *Y*_(NO)_ and *Y*_(NPQ),_ respectively, constructed with 12 increasing values of irradiance. Data shown correspond to the means ± SD of 4 biological replicates

The impact exerted by magnesium shortage conditions on *B. braunii* physiology was also assessed at the level of photosynthetic performance. First, we observed that the *F*_*v*_/*F*_*m*_ values remained stable for all groups during most part of the incubation period (Fig. 6a); nevertheless, it underwent a significant decline at day 23 in cells cultivated with Mg^2+^ 0.0037 g L^−1^. The *F*_*v*_/*F*_*m*_ serves as an indicator of the level of physiological stress (Malapascua et al. 2014), and thus this change reveals that there is a critical point, where magnesium limitation becomes harmful for cell metabolism due to its distribution and dilution among several cell generations. Moreover, at day 23 the same treatment prompted a significant divergence of the energy absorption and utilization as *Y*_(II)_, *Y*_(NPQ)_ and *Y*_(NO)_ differed significantly from those of the remainder treatments (Fig. 6b–d). Interestingly, the effective quantum yield *Y*_(II)_ (Fig. 6b), involved in the electron transport rate (photosynthetic capacity), did not differ among the different levels of MgSO_4_; however, the yield losses, i.e., *Y*_(NO)_ and *Y*_(NPQ)_ presented different patterns according to the level of MgSO_4_. In the range of 0.1104 to 0.0185, *Y*_(NPQ)_ (Fig. 6c) dominated as yield loss, whereas at the lowest level of MgSO_4,_ i.e., 0.0037, *Y*_(NO)_ (Fig. 6d) was the prevalent yield loss. *Y*_(NO)_ is the fraction of energy passively dissipated as heat and fluorescence, mainly due to the closure of PSII reaction centers. High values indicate an inability of the alga to protect itself against photodamage by an excess of radiation (Hendrickson et al. 2004), whereas *Y*_(NPQ)_ is the fraction of energy dissipated as heat via regulated photoprotective mechanisms. High values are indicative of the photoprotection capacity (Kramer et al. 2004). Thus, at high–medium level of MgSO_4,_ the quenching mechanism mainly through *Y*_(NPQ)_ indicates a regulated and high photoprotection cellular state, related to the xanthophyll cycle. In contrast, high *Y*_(NO)_ values under low levels of MgSO_4_ indicated non-photoregulated thermal dissipation of energy, with less photoprotection capacity compared the photoregulated process related to *Y*_(NPQ)_. Stress conditions such as increased UVB radiation or low carbon availability produced an increase in the NPQ_max_ in green alga *Ulva rigida* (Figueroa et al. 2014, 2021). Since NPQ_max_ is the ratio *Y*_(NPQ)_: *Y*_(NO)_, it is shown that stress conditions favored the photoregulated yield loss increasing the photoprotection capacity. In this study, at the latest stages of incubation, the lowest MgSO_4_ level exerted excessive stress on the cells and the dominance of *Y*_(NO)_ at high irradiances compared to *Y*_(NPQ)_-associated loss.

The variations of the proportion of yield losses may be a consequence of a combined effect between the depletion of nitrogen and phosphorous levels at the final stages of growth (Fig. 1b, c) while also considering the severe magnesium-limited conditions, as this cation is crucial for membrane stacking and assembling of protein–pigment complexes in photosystems I and II. Furthermore, a considerable number of enzymes require Mg^2+^ ions for their activation; thus, a substantial number of control points in glycolysis, oxidative phosphorylation, protein and DNA synthesis, and FA metabolism have been shown to be responsive to Mg^2+^ ion availability in vivo (Rubin 1975; Vernon and Wacker 1978; Algranati 1980; Åkerman 1981; Terasaki and Rubin 1985; Grubbs and Maguire 1987; Walker 1994). Therefore, given the difficulty to assimilate carbon for biomass accumulation under such nutritional burden, the cells may respond by increasing the level of non-photochemical dissipation of exitonic energy to cope with the overflow of reductive power (Pessarakli 2016). Likewise, the fact that magnesium deficient cells exhibited major lipid content at the end of the batch (Fig. 1b), led us to hypothesize that even under disadvantageous nutritional conditions for biomass production, *B. braunii* can channel electrons from the electron transfer chain towards highly reduced compounds such neutral lipids as a mechanism to alleviate the harmful reductive pressure at the reaction centers level (Gollan et al. 2017; Burlacot et al. 2019).

Sasaki et al. (1997), demonstrated that acetyl-CoA carboxylase (ACCase), a key multifunctional enzyme complex catalyzing the first committed step of fatty acid synthesis, is regulated by the availability of Mg^2+^ and light energy via thioredoxin. Considering the decrease in the chlorophyll content and the absorption coefficient caused by the low availability of Mg^2+^, a greater light availability for the cells can be expected. According to the results described by the same authors, a higher average irradiance across the culture triggers an increase in the production of reducing power, which in turn leads to an increase in the reduced form of the thioredoxin enzyme. This enzyme is involved in the activation of plastidic ACCase, which could explain an augmented capacity for lipid production as it provides the precursor for FA synthesis malonyl-CoA. Similar results were reported by (Chen et al. 2019). Moreover, Sasaki et al. points out that the activity of ACCase is also dependent on the availability of Mg^2+^, which showed to have an optimal value above which it can become an inhibitory agent for the enzyme.

On the other hand, Volgusheva et al. (2015) found that Mg^2+^-deprived *Chlamydomonas* cells showed higher hydrogen (H_2_) production and still retained a high proportion of active PSII reaction centers. In addition, more than 80% of the electrons used for H_2_ production came from water-splitting in the PSII. In that case, the reducing power is driven towards the formation of H_2_ under anaerobic conditions due to the limitation of inorganic carbon as an electronic sink. This makes it possible to infer that upon Mg^2+^ deficiency, the chloroplast retains the ability to generate reducing power and, therefore, NADPH. On the other hand, these authors suggest that despite the Mg^2+^ limitation, the level of expression and activity of the enzymes related to the electron transport chain in the thylakoid membranes and the RuBisCO enzyme are not drastically affected. In that sense, and considering that in our case the cells were periodically supplied with carbon, we can infer that in addition to the futile cycles (Pessarakli 2016; Nikkanen et al. 2021), highly reduced molecules such as the lipids might serve as an alternative mechanism to alleviate the excess of reducing power due to the high requirement of reducing equivalents for the biosynthesis of these molecules. Just to mention one example, one elongation cycle of a fatty acyl chain performed by the fatty acid synthase (FAS) multisubunit enzyme, requires stoichiometric amount of ATP, acetyl CoA and NADPH for each two carbon units added to the growing acyl chain (Cagliari et al. 2011). Nevertheless, the physiological and metabolic mechanisms by which Mg^2+^ availability is related to the regulation of reducing power partitioning and lipid metabolism in *B. braunii* still remains unclear, thus its elucidation opens a new frontier of research.

## Conclusion

Microalgal cells harbor notable biochemical properties that make them attractive for their exploitation as a renewable source of energy-dense biomass. *B. braunii* exhibits a remarkable capacity to produce high amounts of energy-rich compounds such as lipids. However, its application still depends on future improvements in the overall system productivity. In this study we assessed the Mg^2+^ limitation as an alternative approach to reduce the chlorophyll content of *B. braunii* biomass and improve the light usage, which in turn may potentially enhance the areal productivity in the culture without the need of genetic intervention of the cells. Our results indicated that reducing the availability of this cation not only decreases the pigment and light absorption capacity of this alga, but also induces the accumulation of highly reduced and energy-dense metabolites such as lipids, without affecting cellular growth. Moreover, the changes obtained on these cellular features in the present study represent a starting point for further optimization, since the regulatory role of Mg^2+^ on energy and lipid metabolism is still unclear and its fine tuning in microalgae cell cultures requires a thorough investigation under continuous culture conditions and upon different light regimes.

### Supplementary Information

**Additional file 1: Fig S1.** Thin-layer chromatography plaques photographs. **Fig S2.** Cultures Photographs. **Fig. S3.** Photomicrographs of *B. braunii* cells in DIC and fluorescence.

## Data Availability

The data sets used and/or analyzed during the current study are available from the corresponding author on reasonable request.

## Abbreviations

ANOVA: Analysis of variance
ACCase: Acetyl-CoA carboxylase
ATP: Adenosine triphosphate
ChlA: Chlorophyll *a*
CHOs: Carbohydrates
DAG: Diacylglycerol
DCW: Dry cell weight
DGDG: Digalactosyldiacylglycerol
DGTS: Diacylglyceryl trimethyl homoserine
DIC: Differential contrast interference microscopy
DMSO: Dimethyl sulfoxide
DNA: Deoxyribonucleic acid
F_0_: Measuring light fluorescence
FA: Fatty acid
Fm: Maximal fluorescence
FNR: Ferredoxin NADP^+^ reductase
Fv: Variable fluorescence
GMO: Genetically modified organism
K_a_: Absorption coefficient
LED: Light-emitting diodes
LHC: Light-harvesting complex
MGDG: Monogalactosyldiacylglycerol
NADH: Nicotinamide adenine dinucleotide
NADPH: Nicotinamide adenine dinucleotide phosphate
NPQ_max_: Maximal non-photochemical quenching
OD: Optical density
PC: Phosphatidylcholine
PCA: Principal components analysis
PSII: Photosystem II
RLC: Rapid light curve
SD: Standard deviation
TAG: Triacylglycerol
TLC: Thin-layer chromatography
UPLC–MS: Ultra high-performance liquid chromatography–Mass spectrometry
*Y*_(NO)_: Passive non-photochemical quenching
*Y*_(NPQ)_: Active non-photochemical quenching
*Y*_II_: Effective quantum yield of PSII photochemistry
