# Supplementary material for: Reducing self-shading effects in Botryococcus braunii cultures: effect of Mg2+ deficiency on optical and biochemical properties, photosynthesis and lipidomic profile
Source: Bioresour Bioprocess. 2021 Apr 26;8(1):33. doi: 10.1186/s40643-021-00389-z (PMC10992481; doi:10.1186/s40643-021-00389-z)
Supplement: Supplementary file 1 — Additional file 1: Fig S1. Thin-layer chromatography plaques photographs. Fig S2. Cultures Photographs. Fig. S3. Photomicrographs of B. braunii cells in DIC and fluorescence. [file 40643_2021_389_MOESM1_ESM.docx]

**Fig. S1**. Thin layer chromatography plaques run with total lipid extracts obtained from *B. braunii* cells cultivated for 23 days in media prepared with different concentrations of MgSO_4_.The asterisk and the triangle indicate the hydrocarbon and the carotenoid bands, respectively. The TLC pattern obtained in similar conditions reported by other authors were used as reference (Fang et al. 2015; Moutel et al. 2016)


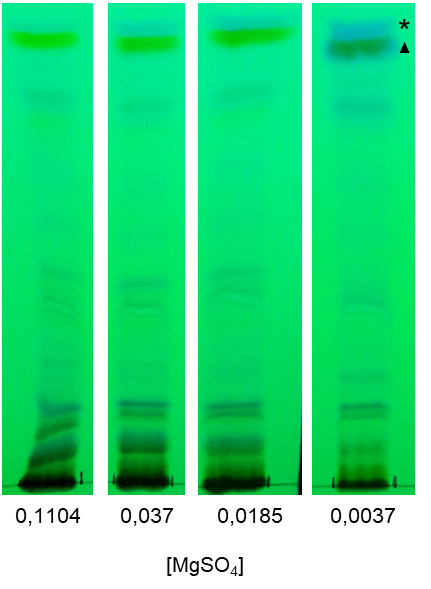

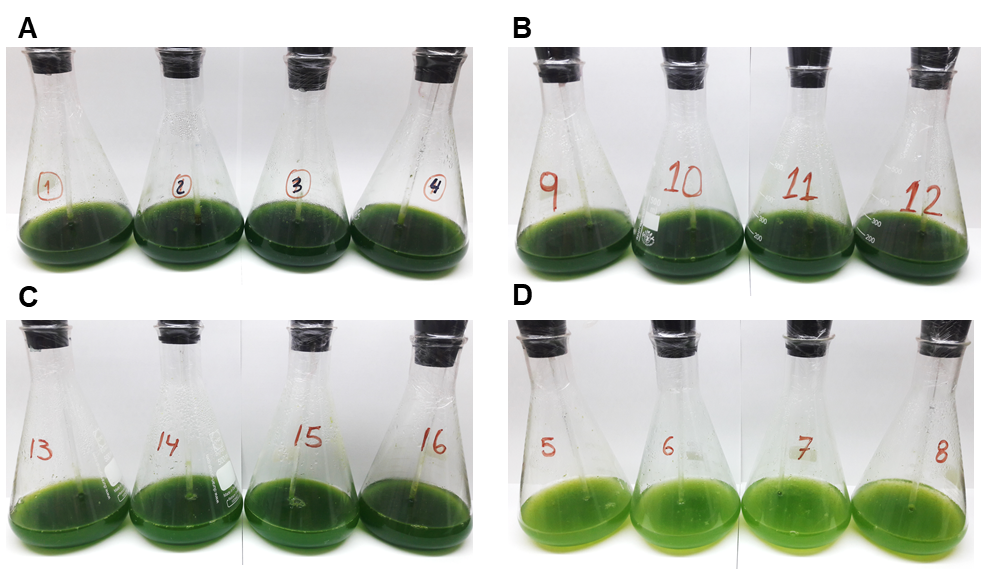


**Fig. S2.** Cultures of *B. braunii* after 23 days of incubation in media prepared with different concentrations of MgSO_4_. (**A)** 0.1104 g L^-1^; (**B**) 0.037 g L^-1^;(**C**) 0.0184 g L^-1^; (**D**) 0.0037 g L^-1^.


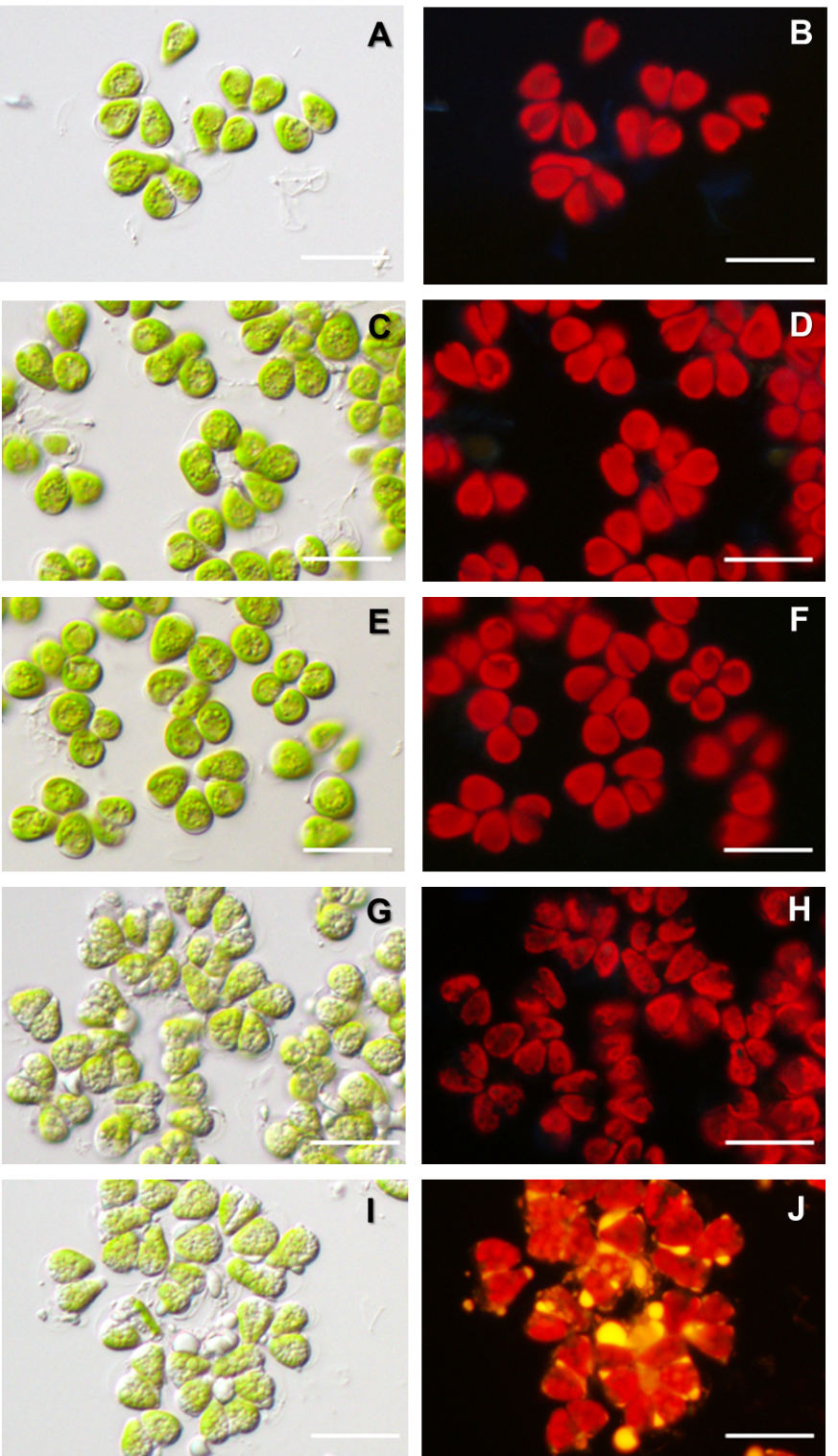


**Fig. S3.** Photomicrographs in Differential Interference Contrast (DIC) (left side) and fluorescence (right side) of *B. braunii* cells sampled after 23 days of incubation in modified BG_11_ medium with different concentrations of MgSO_4_. (**A** and **B**) 0.1104 g L^-1^; (**C** and **D**) 0.037 g L^-1^; (**E** and **F**) 0.0184 g L^-1^; (**G** and **H**) 0.0037 g L^-1^. (**I**) and (**J**) also correspond to 0.0037 g L^-1^ samples. (**J**) was taken from cells stained with Nile Red. The scale corresponds to 20µm.
